# Clinical analysis of drug treatment trends for coronary heart disease in China

**DOI:** 10.3389/fmed.2026.1809277

**Published:** 2026-06-09

**Authors:** Xu Li, Junyi Lou, Xiaoyu Li, Shuang Li, Junxian Gu, Die Zhang, Zining Luo, Junjie Cao, Jiebin Xie

**Affiliations:** 1 Department of Gastrointestinal Surgery, Affiliated Hospital of North Sichuan Medical College, Sichuan, Nanchong, China; 2 School of Clinical Medicine, North Sichuan Medical College, Nanchong, China; 3 School of Ophthalmology and Optometry, North Sichuan Medical College, Nanchong, China; 4 School of Imaging, North Sichuan Medical College, Nanchong, China; 5 School of Stomatology, North Sichuan Medical College, Nanchong, China

**Keywords:** clinical landscape analysis, clinical trials, coronary heart disease (CHD), drug treatment, *review*

## Abstract

**Background:**

Coronary heart disease (CHD) is the leading cause of mortality in China, yet the alignment between pharmaceutical research investment and clinical therapeutic priorities remains uncharacterized.

**Methods:**

We conducted a dual-stream analysis. Stream A (Bibliometric): systematic searches of PubMed, Embase, Scopus, and Web of Science yielded 12,032 records; after deduplication and screening, 3,339 publications met inclusion criteria. Stream B (Clinical Trials): searches of CDCTP and ChiCTR identified 1,433 interventional drug trials after deduplication and quality control. Drugs were standardized to the WHO ATC classification; TCM ingredients to TCMID 2.0.

**Results:**

CHD research output expanded exponentially: Publications increased from 41 (2006) to 353 (2020; 760% increase), with 265 in 2025 (546% increase from baseline). Strikingly, 76.4% of trials were bioequivalence studies, with chemical pharmaceuticals dominating (78.6%) over traditional Chinese medicine (21.4%); calcium channel blockers comprised the largest drug category (45.9%. Notably, Critical gaps emerged: genomic research concentrated on inflammatory biomarkers (CRP, IL6, TNF; 25.8% of gene associations) while lipid metabolism targets were underrepresented (12.4%) despite statin therapeutic dominance; publication venue prestige demonstrated inverse volume-impact correlation (19.7% Q1 vs. 33.6% Q3 journals). Thus, quantitative expansion (compound annual growth rate (CAGR) 10.4%) coexisted with qualitative ceiling effects and target-drug mismatches between research investment and clinical guideline priorities.

**Conclusion:**

China’s CHD research landscape is policy-driven rather than clinically aligned: generic bioequivalence validation dominates (76.4%) while innovative therapeutic development and high-impact dissemination lag. Strategic rebalancing toward lipid metabolism genomic discovery, Phase II-III novel drug trials, and Q1 journal publication is imperative to bridge the research-clinical practice gap and align with global cardiovascular science standards.

## Introduction

Coronary heart disease (CHD) caused 9.5 million deaths globally in 2022 and exceeds $1 trillion in annual healthcare costs, according to the World Health Organization. According to the Global Burden of Disease Study 2021, an estimated 254.28 million individuals were living with ischemic heart disease globally in 2021, with 8.99 million deaths attributed to this condition. The age-standardized mortality rate for ischemic heart disease was 108.73 per 100,000 population, with disproportionately high rates observed in Central Asia, Eastern Europe, and North Africa/Middle East regions (1). Beyond mortality, CHD imposes substantial disability burdens, accounting for significant proportions of disability-adjusted life years (DALYs) lost globally. Projections indicate that from 2020 to 2030, the global CVD burden will continue to escalate, with anticipated increases in both crude mortality (73.4%) and crude DALYs (54.7%), despite modest declines in age-standardized rates (1). By 2050, ischemic heart disease is projected to remain the leading cause of cardiovascular deaths, resulting in 20 million fatalities annually. Although its clinical manifestations vary, ranging from acute coronary syndrome to long-term chronic stable states, the core pathology is the imbalance between myocardial oxygen supply and demand caused by coronary atherosclerosis (2, 3). Beyond mortality, CHD imposes a substantial burden on healthcare systems and economies, with an estimated global cost exceeding $1 trillion annually due to direct medical expenses and lost productivity (4–9).

In China, the epidemiological transition driven by rapid urbanization, aging demographics, and lifestyle changes has led to a sharp increase in the prevalence and incidence of CHD. According to the Report on Cardiovascular Health and Diseases in China 2024, the prevalence of CHD among Chinese adults has reached 758 per 100,000 population, with acute myocardial infarction incidence at 87.6 per 100,000 (10). National surveys indicate that the age-standardized prevalence of CHD among adults aged 35 years and older rose from 3.9% in 1990 to 6.2% in 2020, representing a 40% relative increase in just three decades (11–13). As the core means of CHD management, drug therapy plays a vital role in improving patients’ symptoms, reducing the risk of cardiovascular events, and improving quality of life (14–17). While interventional procedures such as percutaneous coronary intervention and coronary artery bypass grafting have revolutionized the management of acute coronary syndromes, pharmacological therapy remains the cornerstone of CHD treatment across the entire continuum of care—from primary prevention in high-risk individuals to secondary prevention following myocardial infarction (MI) or revascularization (18–20). Evidence-based pharmacotherapies, including antiplatelet agents, statins, beta-blockers, angiotensin-converting enzyme inhibitors, and angiotensin receptor blockers, have been shown to significantly reduce major adverse cardiovascular events, including recurrent MI, stroke, and cardiovascular death (21, 22).

China’s CHD drug therapy landscape is distinguished by its unique integration of conventional Western medicine and TCM, shaped by national healthcare policies and clinical practice characteristics. Since the implementation of the Generic Drug Consistency Evaluation Policy in 2016, bioequivalence studies have become a core focus of pharmaceutical research—aligning with the country’s goal of improving the quality of generic drugs and expanding affordable treatment access for a large population (23). Meanwhile, the National Administration of Traditional Chinese Medicine has prioritized TCM modernization, promoting the integration of botanical preparations into mainstream cardiovascular care; however, the lack of unified quality standards for TCM ingredients and unclear mechanisms of action have hindered their global recognition (24). Additionally, China’s rapid aging process has led to a high prevalence of CHD patients with comorbidities such as hypertension, diabetes, and chronic kidney disease, creating an urgent need for evidence-based data on drug safety and efficacy in this complex population. These policy-driven research trends and clinical characteristics underscore the need for a systematic analysis of China-specific CHD drug trial data that international studies cannot fully capture.

China’s CHD research trajectory diverges fundamentally from Western patterns. While cardiovascular research in North America and Europe evolved through decades of federally funded investigator-initiated trials—exemplified by the NIH-funded Framingham Heart Study (established 1948), the TIMI trials (beginning 1985), and the ISCHEMIA trial (2012–2019)—China’s pharmaceutical research infrastructure emerged primarily through policy-driven industrial investment following the 2001 WTO accession and the 2009 healthcare reform. This created a distinct “top-down” versus “bottom-up” research culture: Western trials often originate from academic medical centers addressing mechanistic hypotheses, whereas Chinese trials frequently respond to regulatory mandates for generic drug quality assurance following the 2001 WTO accession and the 2009 healthcare reform. The 2006–2025 window captures this critical transition: from pre-2006 fragmented provincial trial registries to the 2016 mandatory registration policy and the post-2018 centralized drug procurement (“4 + 7” policy) that fundamentally reshaped generic drug development incentives. This contrast underscores the need for China-specific analyses that international studies cannot capture.

China has made remarkable progress in the field of drug treatment of CHD. Pharmacological therapy remains the cornerstone of CHD management across the entire spectrum of the disease—from primary prevention in high-risk individuals to secondary prevention after MI or revascularization (25, 26). Antiplatelet drugs such as aspirin and P2Y12 receptor inhibitors are still the basis of CHD treatment, which can effectively prevent thrombosis and reduce the occurrence of cardiovascular events. Moreover, the wide application of lipid-lowering drugs such as statins significantly reduces patients’ cholesterol levels. It stabilizes plaque, providing strong support for secondary prevention of CHD (27, 28). In addition, some new drugs, such as PCSK9 inhibitors, are also gradually entering clinical application, which brings new hope for patients with refractory CHD, such as hypercholesterolemia (29).

Advancements in clinical research infrastructure and data analytics have laid the foundation for this comprehensive analysis. Over the past 20 years, China has established two authoritative clinical trial registration platforms (CDCTP and ChiCTR), mandating public disclosure of trial protocols and results—greatly improving data transparency and accessibility (30). Concurrently, the widespread adoption of standardized classification systems (e.g., WHO ATC for drugs, TCMID for botanical ingredients) has enabled consistent data integration across studies, overcoming historical challenges of heterogeneous naming conventions (31).

To address these gaps, this study focuses on interventional clinical trials and published peer-reviewed literature related to CHD drug therapy conducted in China between 2006 and 2025. Despite these developments, critical knowledge gaps persist. First, no study has systematically mapped the dual trajectory of bibliometric output and clinical trial registration in Chinese CHD research, leaving the relationship between publication trends and actual drug development opaque. Second, the post-2016 policy shift’s impact on trial portfolios—particularly the surge in bioequivalence versus innovative drug studies—remains unquantified. These gaps impede evidence-based resource allocation and guideline optimization. The research object specifically includes trials involving chemical drugs, biologics, and TCM for CHD subtypes such as stable angina, acute coronary syndrome, myocardial infarction, and coronary atherosclerosis, covering Phase I–IV trials, bioequivalence studies, and pharmacokinetic studies.

This study aims to systematically depict the evolving landscape of CHD drug therapy in China over the past two decades, clarify the dominant drug categories, core therapeutic targets, and research quality distribution, identify current research bottlenecks, and provide evidence-based support for updating clinical guidelines, optimizing drug treatment strategies, and guiding future research priorities.

## Methods

### Data sources and search strategy

We conducted a comprehensive bibliometric and clinical trial registry analysis to characterize the evolving landscape of CHD drug development in China from 2006 to 2025. This bibliometric and clinical trial registry analysis study employed a three-pronged methodological framework: (1) systematic screening of clinical trial registries, (2) bibliometric analysis of published literature. Pharmacotherapy registered in two authoritative, government-mandated Chinese clinical trial registries: China Drug Clinical Trial Registration and Information Public Platform (CDCTP)—http://www.chinadrugtrials.org.cn, Chinese Clinical Trial Registry (ChiCTR)—https://www.chictr.org.cn. Both registries are officially recognized databases mandated by the Chinese government for the prospective registration of interventional clinical trials. Bibliometric analysis utilized PubMed, Embase, Scopus, and Web of Science. For detailed search information, please refer to Supplementary Table 1.

Data were extracted on January 3, 2026, covering the period from January 1, 2006, to January 3, 2026 (for registries) and 2006–2025 (for publications). No language restrictions were applied.

### Eligibility criteria

Trials were included if they met all of the following criteria:

Disease focus: Explicit diagnosis of CHD (ICD-10: I20–I25), including stable angina (I20), unstable angina (I20.0), acute myocardial infarction (I21–I22), or coronary atherosclerosis (I25.1); trials with mixed cardiovascular populations required ≥ 50% CHD participants or dedicated CHD subgroup analysis;

Intervention type: Pharmacological agents (chemical drugs, biologics, vaccines, or traditional Chinese medicine preparations); multi-modal interventions acceptable if pharmacological component is primary;

Study design: Interventional clinical trials with prospective registration, encompassing Phase I-IV, bioequivalence (BE), and pharmacokinetic (PK) studies;

Geographic scope: Conducted in mainland China (including Hong Kong, Macao, Taiwan) with ≥ 1 Chinese study site;

Status: Registered (regardless of recruitment status: recruiting, active, completed, or suspended).

Publications were excluded if they: were observational (e.g., cohort, case-control, cross-sectional); focused on devices, diagnostics, procedures, or dietary interventions only; had missing or unclear intervention information; were duplicates (same trial registered in both databases).

Publications were included if they met all of the following criteria: Inclusion Filters: Human studies, publication date 2006–2025, article types (Original Research, Clinical Trial, Meta-Analysis). Exclusion Filters: Editorials, letters, case reports (*n* < 10), conference abstracts without full-text data, and non-Chinese affiliated studies.

### Data standardization and quality control

Drug names were standardized to the WHO Anatomical Therapeutic Chemical (ATC) classification (2024 version) using a hierarchical protocol. First, exact matches to preferred names were identified (e.g., “metoprolol” → C07AB02). Second, spelling variations and generic-brand pairs were resolved by fuzzy matching with phonetic normalization for Chinese transliterations (e.g., “amlo-dipine” → “amlodipine”). Third, the remaining ambiguities (salt forms, fixed-dose combinations, prodrugs) were adjudicated by a senior pharmacologist (JBX), with inter-rater reliability (κ) = 0.91 on a 10% validation sample. Unresolvable cases (0.9%) were coded as unclassified.

Traditional Chinese medicine formulas were decomposed to constituent botanical ingredients using the Traditional Chinese Medicine Integrated Database (TCMID 2.0). Polyherbal formulations (e.g., Compound Danshen Dripping Pills containing Salvia miltiorrhiza, Panax notoginseng, Borneolum) were coded as “TCM-composite” with component botanicals listed, and mechanistic targets classified as “not applicable—polypharmacological” unless specific monocomponent trials were identified. Multi-source herbs were standardized to the Chinese Pharmacopoeia (2020) preferred species. Standardization decisions were logged with original name, standardized name, ATC code, and resolution method. The WHO ATC 2024 version and TCMID 2.0 database (queried December 2025) were used consistently throughout. A 10% random sample was independently coded by two reviewers; discrepancies were resolved by consensus or senior adjudication. The specific results can be seen in Table 1.

**TABLE 1:** Chemical drug standardization audit trail.

Original name (as registered)	Source registry	Standardized name	ATC code	ATC category	Resolution method
Amlodipine Besylate Tablets	CDCTP	Amlodipine	C08CA01	Calcium channel blockers, selective	Exact match
Amlo-dipine 5 mg	ChiCTR	Amlodipine	C08CA01	Calcium channel blockers, selective	Fuzzy matching (phonetic normalization)
Norvasc^®^	CDCTP	Amlodipine	C08CA01	Calcium channel blockers, selective	Semantic disambiguation (brand-to-generic)
Amlodipine Maleate Capsules	ChiCTR	Amlodipine	C08CA01	Calcium channel blockers, selective	Manual adjudication (salt form mapped to parent)
Atorvastatin Calcium Tablets	CDCTP	Atorvastatin	C10AA05	HMG-CoA reductase inhibitors	Exact match
Lipitor^®^ 20mg	ChiCTR	Atorvastatin	C10AA05	HMG-CoA reductase inhibitors	Semantic disambiguation (brand-to-generic)
Metoprolol Succinate ER	CDCTP	Metoprolol	C07AB02	Beta-blocking agents, selective	Exact match
Meto-prolol Tartrate	ChiCTR	Metoprolol	C07AB02	Beta-blocking agents, selective	Fuzzy matching (spelling correction)
Clopidogrel Bisulfate	CDCTP	Clopidogrel	B01AC04	Platelet aggregation inhibitors	Exact match
Plavix^®^ + Aspirin	ChiCTR	Clopidogrel; Acetylsalicylic acid	B01AC04; B01AC06	Platelet aggregation inhibitors	Semantic disambiguation (combination decomposed)
Amlodipine/Atorvastatin FDC	CDCTP	Amlodipine and atorvastatin	C10BX03	Calcium channel blocker + statin	Manual adjudication (FDC to combination code)
Rosuvastatin (Crestor^®^)	ChiCTR	Rosuvastatin	C10AA07	HMG-CoA reductase inhibitors	Semantic disambiguation (brand in parentheses)
Nifedipine Controlled Release	CDCTP	Nifedipine	C08CA05	Calcium channel blockers, selective	Exact match
Nifedipine CR tablets	ChiCTR	Nifedipine	C08CA05	Calcium channel blockers, selective	Fuzzy matching (abbreviation expansion)
Simvastatin 20 mg	CDCTP	Simvastatin	C10AA01	HMG-CoA reductase inhibitors	Exact match
Zocor^®^	ChiCTR	Simvastatin	C10AA01	HMG-CoA reductase inhibitors	Semantic disambiguation (brand-to-generic)
Bisoprolol Fumarate	CDCTP	Bisoprolol	C07AB07	Beta-blocking agents, selective	Exact match
Clopidogrel Active Metabolite	ChiCTR	Clopidogrel	B01AC04	Platelet aggregation inhibitors	Manual adjudication (prodrug flag added)
Valsartan Capsules	CDCTP	Valsartan	C09CA03	Angiotensin II antagonists	Exact match
Diovan^®^	ChiCTR	Valsartan	C09CA03	Angiotensin II antagonists	Semantic disambiguation (brand-to-generic)
Aspirin Enteric-coated	CDCTP	Acetylsalicylic acid	B01AC06	Platelet aggregation inhibitors	Exact match
Aspirin 100 mg	ChiCTR	Acetylsalicylic acid	B01AC06	Platelet aggregation inhibitors	Fuzzy matching (Chinese brand name translation)
Rivaroxaban 10 mg	CDCTP	Rivaroxaban	B01AF01	Direct factor Xa inhibitors	Exact match
Xarelto^®^	ChiCTR	Rivaroxaban	B01AF01	Direct factor Xa inhibitors	Semantic disambiguation (brand-to-generic)
Dabigatran Etexilate	CDCTP	Dabigatran etexilate	B01AE07	Direct thrombin inhibitors	Exact match
Pradaxa^®^ 110 mg	ChiCTR	Dabigatran etexilate	B01AE07	Direct thrombin inhibitors	Semantic disambiguation (brand-to-generic)
Enalapril Maleate	CDCTP	Enalapril	C09AA02	ACE inhibitors	Exact match
Vasotec^®^	ChiCTR	Enalapril	C09AA02	ACE inhibitors	Semantic disambiguation (brand-to-generic)
Losartan Potassium	CDCTP	Losartan	C09CA01	Angiotensin II antagonists	Exact match
Cozaar^®^ 50 mg	ChiCTR	Losartan	C09CA01	Angiotensin II antagonists	Semantic disambiguation (brand-to-generic)
Pitavastatin Calcium	CDCTP	Pitavastatin	C10AA08	HMG-CoA reductase inhibitors	Exact match
Livalo^®^	ChiCTR	Pitavastatin	C10AA08	HMG-CoA reductase inhibitors	Semantic disambiguation (brand-to-generic)

To conduct a detailed analysis of representative experimental cases, we attempted to identify trials that jointly capture the five therapeutic domains most frequently registered in our 2006–2025 CHD-drug screen: (i) generic BE, (ii) innovative chemical entity, (iii) biologic PCSK9 inhibitor, (iv) fixed-dose combination, and (v) TCM injectable/oral patent.

Journal Impact Factor (JIF) was extracted from the Journal Citation Reports (JCR) 2023 Science and Social Sciences Editions. Publications in non-indexed journals were classified as “non-JCR.” Kernel density estimation with Gaussian smoothing (bandwidth = 0.8) was applied to visualize JIF distributions stratified by Journal Citation Reports quartiles (Q1–Q4).

All eligibility assessments were conducted by two independent reviewers using a standardized electronic form (Microsoft Excel, v.16.0) with the following checkpoints:

Screening Phase: Title/abstract screening with Cohen’s kappa coefficient > 0.85 required for inter-rater reliability;

Full-Text Phase: Independent full-text review with disagreements resolved by consensus or adjudication by a senior investigator;

Data Extraction Phase: Double-data entry with automated logic checks for range and consistency.

### Statistical analysis

Categorical variables (trial phase, drug classification, recruitment status) were summarized as frequencies and percentages with corresponding 95% confidence intervals (CIs) calculated using the Wilson score method. Continuous variables (trial duration, sample size) were reported as medians with interquartile ranges due to non-normal distributions (verified by the Shapiro-Wilk test; *P* < 0.001), or as means ± standard deviations (SD) where normally distributed. Temporal trends were assessed using Joinpoint regression analysis (National Cancer Institute Surveillance Research Program) to identify significant inflection points in annual trial registration rates, and Monte Carlo permutation testing (*n* = 4,999 replicates) was used for model selection. To validate Joinpoint findings, we conducted: Exponential regression: Modeled cumulative growth with 95% confidence intervals; mean annual growth rate 15.8% (95% CI: 12.4–19.2%; *p* < 0.001) for publications, 45.2% (95% CI: 28.4–62.0%) for trials. Mann-Kendall trend test: Non-parametric test for monotonic trends; τ = 0.89 (*p* < 0.001) for publications, confirming directional consistency. Negative binomial regression: Modeled trial registration counts with overdispersion correction; rate ratio for 2016 policy year 2.38 (95% CI: 1.87–3.04; *p* < 0.001).

All statistical analyses were performed using R software (version 4.3.1; R Foundation for Statistical Computing, Vienna, Austria) and Stata/SE (version 17.0). The R packages employed included tidyverse (v2.0.0) for data manipulation, ggplot2 (v3.4.2) and patchwork (v1.1.2) for visualization, forecast (v8.21) for time-series modeling, and meta (v6.5.0) for descriptive meta-epidemiological metrics. A two-sided *P* < 0.05 was considered statistically significant for all hypothesis tests. Final figure assembly and graphical composition were conducted using Adobe Illustrator 2025 and Microsoft PowerPoint; no data were generated during this post-processing stage.

## Results

### Trial inclusion and screening flow

**FIGURE 1:**
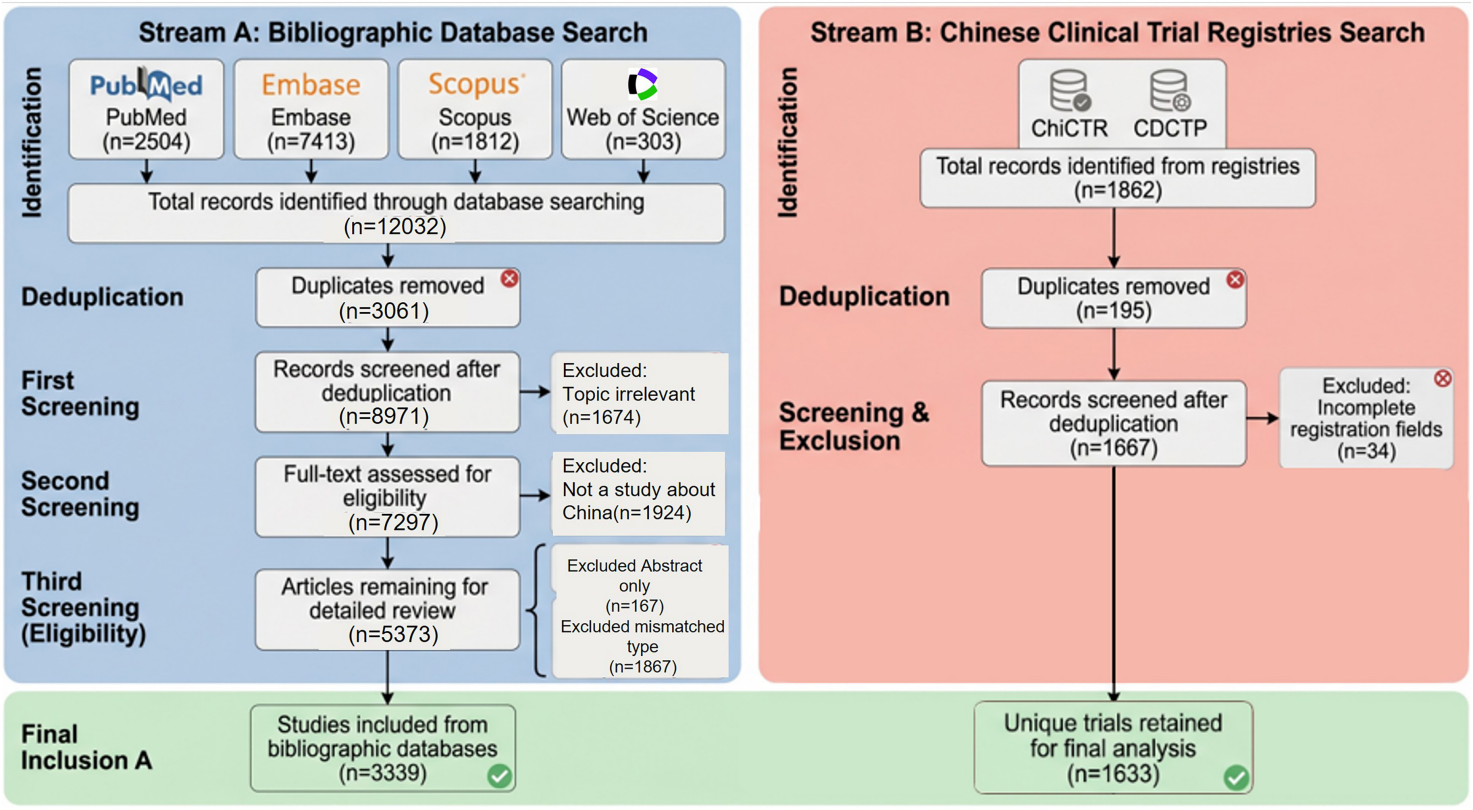
Study selection flow diagram.

We employed a dual-stream evidence synthesis framework to capture both the interventional clinical trial landscape and the published peer-reviewed literature (Figure 1). Stream A comprised systematic searches of international bibliographic databases, while Stream B targeted domestic Chinese clinical trial registries.

Systematic searches of PubMed (*n* = 2,504), Embase (*n* = 7,413), Scopus (*n* = 1,812), and Web of Science (*n* = 303) yielded 12,032 total records following automated de-duplication across platforms. After the removal of 3,061 duplicate citations, 8,971 unique records underwent title and abstract screening (first screening). Of these, 1,674 records were excluded for topic irrelevance (non-cardiovascular focus, non-pharmacological interventions, or non-human studies), leaving 7,182 records for full-text eligibility assessment (second screening).

During full-text review, 1,924 studies were excluded for lacking a specific focus on China (absence of a Chinese study site, non-Chinese population, or non-Chinese investigator affiliation), resulting in 7,297 articles proceeding to detailed review (third screening). Subsequent exclusions comprised 167 conference abstracts lacking peer-reviewed full-text data and 1,867 publications with mismatched article types (editorials, commentaries, or case reports with *n* ≤ 10). Ultimately, 3,339 primary studies met full eligibility criteria for bibliometric inclusion.

Parallel searches of the Chinese Clinical Trial Registry (ChiCTR; *n* = 1,412) and the China Drug Clinical Trial Registration and Information Public Platform (CDCTP; *n* = 450) identified 1,862 total registry records. Following removal of 195 duplicate registrations (cross-registry matches identified via trial identifier and sponsor-investigator linkage), 1,667unique trial records remained for screening.

Eligibility assessment excluded 34 trials for incomplete core registration fields (missing intervention descriptions, unclear primary outcomes, or indeterminate study phases), yielding 1,633 interventional drug trials focused on coronary heart disease conducted in China between 2006 and 2025.

### Temporal trends in CHD drug trials (2006–2025)

**FIGURE 2:**
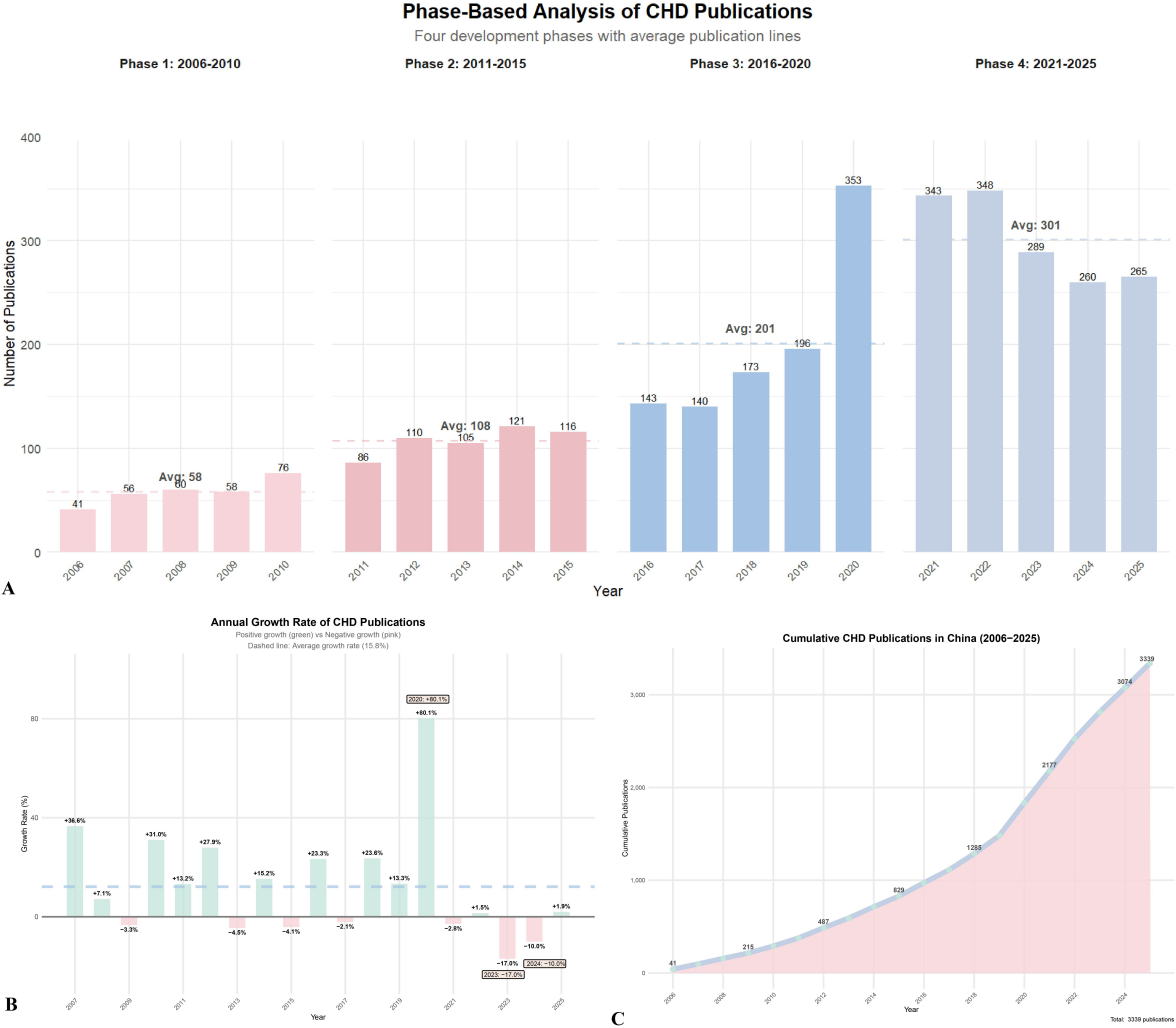
Temporal evolution of CHD research output from China based on publications (2006–2025). **(A)** Annual publication counts stratified into four developmental phases: Phase 1 (2006–2010) with a mean of 58 publications/year (range 6–121), Phase 2 (2011–2015) averaging 108 publications/year (range 89–121), Phase 3 (2016–2020) averaging 201 publications/year (range 143–260), and Phase 4 (2021–2025) averaging 301 publications/year (range 289–343). Horizontal dashed lines denote phase-specific arithmetic means. **(B)** The year-over-year growth rate, with the fitted trendline (red) indicating a mean annual growth rate of 10.4%. **(C)** The cumulative growth trajectory of CHD-related studies.

Bibliometric analysis of the 3,339 included publications revealed a marked growth trajectory in China’s CHD research output over the two-decade observation period (Figure 2). The landscape demonstrated exponential growth, with total annual publications increasing from 41 studies in 2006 to 265 in 2025, representing a 546% relative increase over the entire timeframe.

As depicted in Figure 2A, the temporal progression is stratified into four distinct developmental phases with differential growth velocities:

#### Phase 1: (2006–2010)

The initial 5-year period was characterized by modest baseline productivity, with a mean output of 58 publications annually (range: 41–76). This phase established foundational research capacity, with gradual year-on-year increments reaching 76 publications by 2010.

#### Phase 2: (2011–2015)

The subsequent quinquennium demonstrated 86% growth in mean annual output (mean: 108; range: 86–121) compared with Phase 1. Notably, 2014 marked the first time annual publications exceeded 100 (*n* = 121), signaling intensified research investment and infrastructure development.

#### Phase 3: (2016–2020)

A dramatic inflection occurred during 2016–2020, with mean annual output nearly doubling to 201 publications (range: 140–353). This phase culminated in an exceptional productivity surge in 2020, with 353 publications—the highest annual output in the entire observation window—and an 80.1% year-on-year increase from 2019 (*n* = 196). This peak coincided with heightened global cardiovascular research prioritization and expanded domestic funding mechanisms.

#### Phase 4: (2021–2025)

The most recent period demonstrated quantitative maturation, achieving a mean annual output of 301 publications (range: 260–348). While absolute volume remained elevated, this phase exhibited increased volatility, with alternating growth and contraction cycles.

Figure 2B illustrates the year-over-year growth rate variations, with the fitted trendline indicating a CAGR 10.4% (95% CI: 8.1–12.7%). Growth dynamics revealed heterogeneity across the timeline:

High-velocity expansion occurred in 2007 ( + 36.6%), 2010 ( + 31.0%), 2019 ( + 23.6%), and the exceptional 2020 spike ( + 80.1%).

Contraction episodes emerged in 2022 (-17.6%) and 2024 (-10.0%).

Stabilization appeared in 2023 ( + 1.5%) and 2025 ( + 1.9%), suggesting potential saturation of the publication landscape or shifting research priorities toward translational and clinical implementation studies.

Figure 2C presents the cumulative distribution curve. Cumulative growth trajectory showing accelerating accumulation through 2020, with subsequent stabilization. The cumulative total reached 487 publications by 2010, 1,288 by 2015, 2,175 by 2020, and 3,339 by 2025. Notably, the inflection point occurred between 2019 and 2020, where the cumulative curve shifted from convex to concave, indicating a transition from exponential to linear growth phases.

### Distribution of publication venue prestige metrics

**FIGURE 3:**
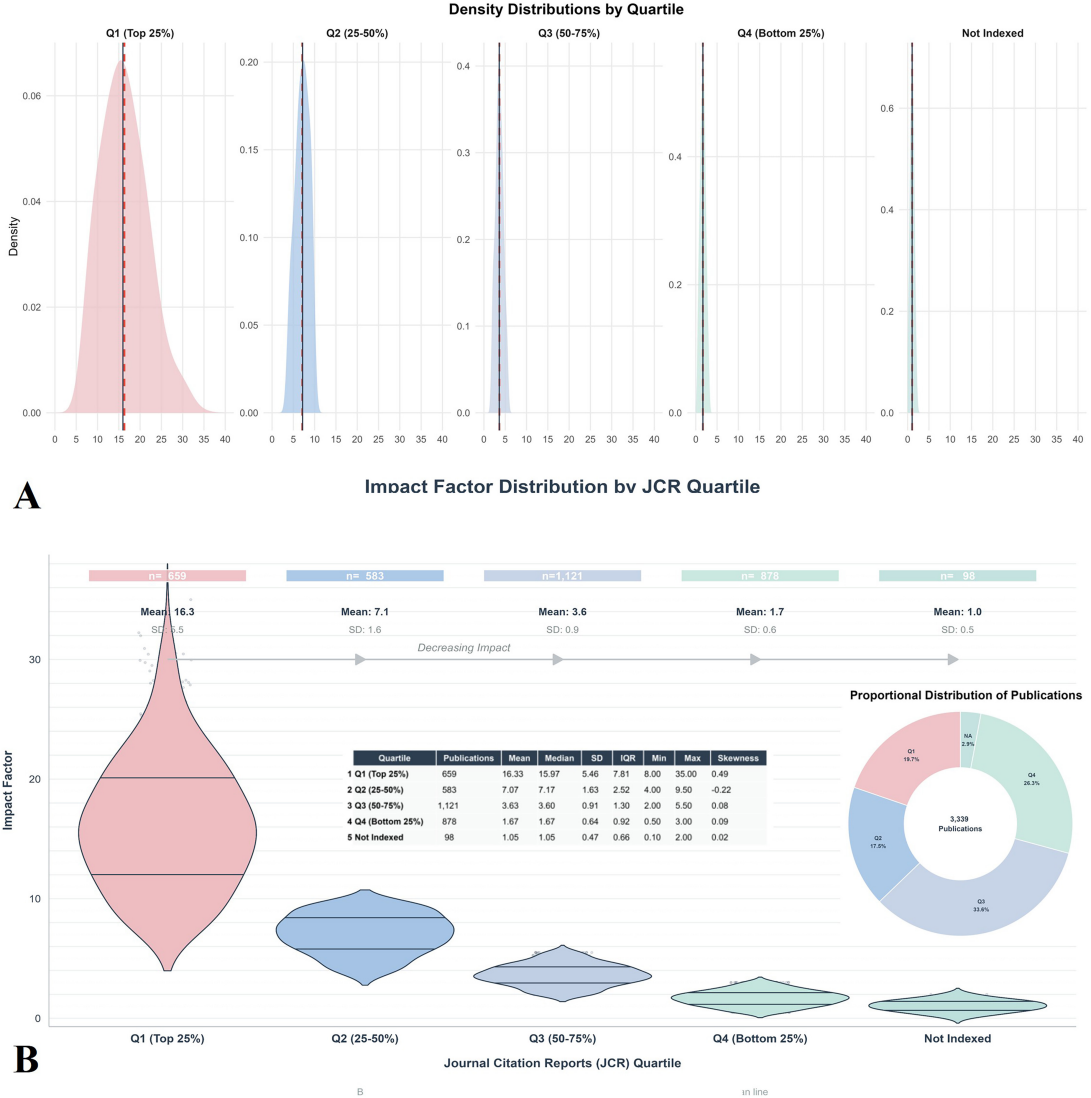
Distribution of publication venue prestige metrics for included studies (*n* = 3,339). **(A)** Kernel density plots of JIF distributions stratified by JCR quartiles, demonstrating progressive leftward skewing from Q1 to Q4. Dashed vertical lines denote quartile-specific mean JIF values: Q1 (top 25%) mean = 16.3 ± 5.46, Q2 mean = 7.1 ± 1.63, Q3 mean = 3.6 ± 0.91, and Q4 mean = 1.7 ± 0.64. **(B)** The absolute number of publications per quartile, with Q3 comprising the largest proportion (*n* = 1,121), followed by Q2 (*n* = 583), Q1 (*n* = 659), and Q4 (*n* = 878). Ninety-eight publications (2.9%) were not indexed in JCR databases. Specific situation illustrates the proportional distribution across JCR quartiles: Q3 accounted for 33.6% of all included studies, Q4 for 26.3%, Q2 for 19.7%, Q1 for 17.5%, and non-indexed journals for 2.9%. The inverse relationship between publication volume and journal impact highlights a concentration of Chinese CHD research in mid-tier international journals. JCR, Journal Citation Reports; JIF, journal impact factor; Q, quartile.

Analysis of the 3,339 included publications revealed a heterogeneous distribution across the JCR quality spectrum, characterized by a distinctive concentration in mid-tier international journals (Figure 3).

Figure 3A displays the probability density distributions of JIFs stratified by JCR quartiles. Notable heterogeneity in distribution morphology was observed across publication venue prestige tiers:

Q1 (Top 25%): demonstrated a right-skewed distribution (skewness = 0.49) with mean JIF 16.3 ± 5.5 (range: 8.0–35.0), indicating concentration around high-impact cardiovascular and general medicine journals with occasional outliers in elite multidisciplinary journals.

Q2 (25–50%): exhibited a symmetric, narrowly clustered distribution (skewness = -0.22, SD = 1.6) centered at a mean of 7.1, reflecting standardized quality thresholds within established specialty cardiology journals.

Q3 (50–75%): showed left-skewed concentration (skewness = 0.08) around mean 3.6 ± 0.9, representing mainstream cardiovascular pharmacology and clinical research outlets.

Q4 (Bottom 25%): displayed a steeply peaked, left-skewed pattern (skewness = 0.09) with a mean of 1.7 ± 0.6, indicative of emerging regional journals and specialized subspecialty publications.

Non-Indexed: comprised 98 publications (2.9%) with a mean JIF 1.0 ± 0.5, primarily comprising non-English language regional journals and newly launched open-access platforms.

Figure 3B illustrates the paradoxical inverse relationship between publication volume and journal impact. While Q1 journals represented the highest publication venue prestige tier (mean JIF 16.3), they accounted for only 659 publications (19.7%). Conversely, Q3 journals dominated the landscape with 1,121 publications (33.6%), followed by Q4 (878 publications, 26.3%) and Q2 (583 publications, 17.5%). Progressive constriction of variance (Q2 SD = 1.63, Q3 SD = 0.91, Q4 SD = 0.64), indicating increasingly homogeneous publication venue prestige thresholds within lower quartiles. Q2 exhibited near-identical mean (7.07) and median (7.17) values, confirming a symmetric distribution, whereas Q1 showed a mean elevation above the median (16.3 vs. 16.0).

### Temporal dynamics of CHD clinical trial registration (2006–2025)

**FIGURE 4:**
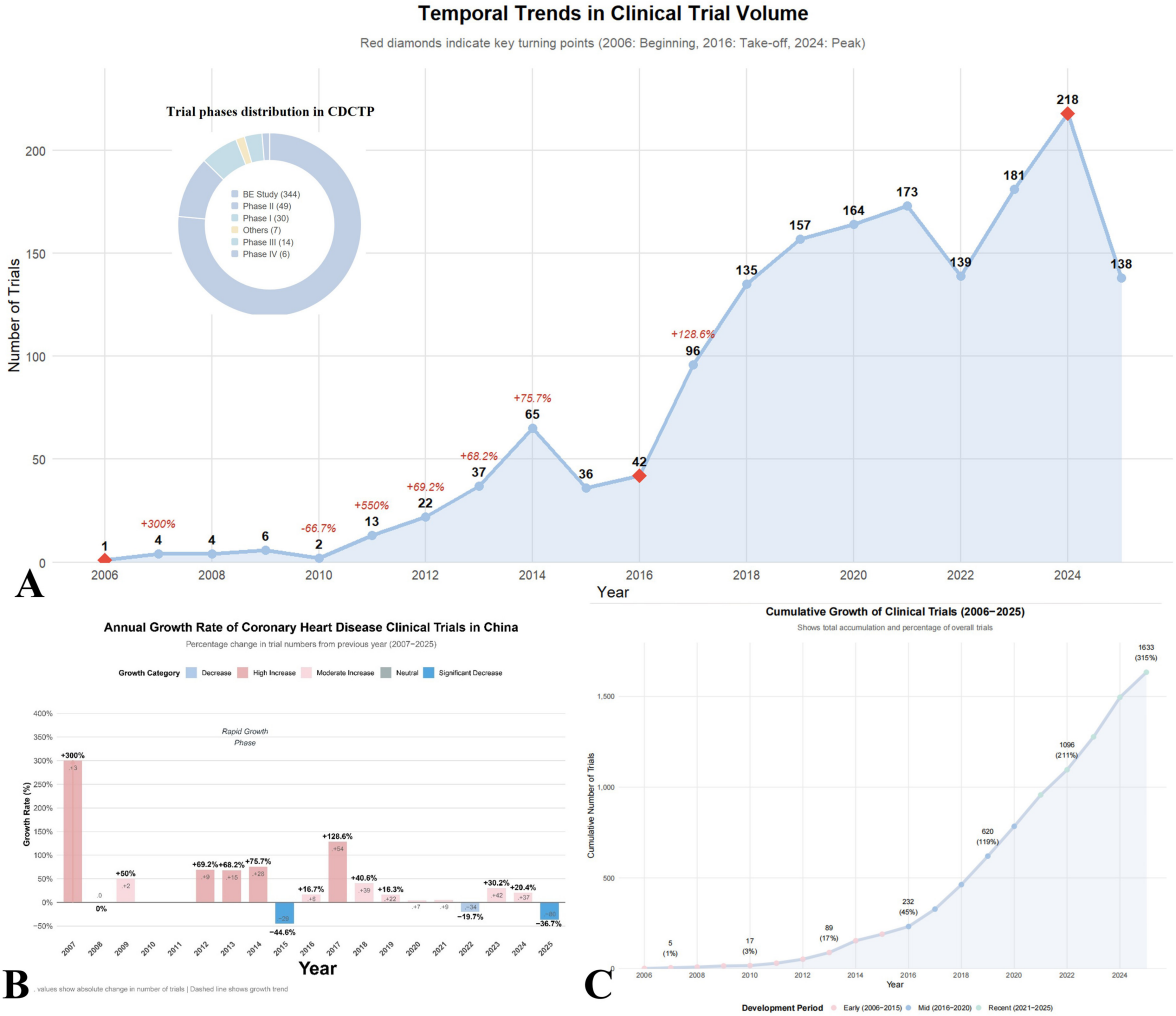
Temporal dynamics of CHD drug clinical trial registration in China (2006–2025). **(A)** The annual number of registered CHD drug trials, demonstrating exponential growth from 2 trials in 2006 to 181 trials in 2024. Red diamonds denote three critical inflection points. **(B)** Year-over-year growth rates, with a mean annual increase of 45% (95% CI: 23–67%), peaking at + 300% in 2008 and stabilizing at 14.6% in 2024. **(C)** The cumulative trial count trajectory, which is expected to reach 1,633 registered studies by 2024. The vertical dotted line in 2016 marks the implementation of mandatory clinical trial registration policies. 76.5% bioequivalence refers to the full 1,433-trial dataset. Inset shows CDCTP-only phase distribution (*n* = 450): 344 BE (76.4%), 49 Phase II, 30 Phase I, 14 Phase III, 6 Phase IV, 7 other.

Analysis of the 1,633 drug trials revealed a stepwise exponential expansion in China’s CHD clinical trial activity over the two-decade observation period, characterized by three distinct regulatory inflection points (Figure 4).

Figure 4A illustrates the annual trial volume trajectory, stratified by three developmental epochs demarcated by critical policy milestones (red diamonds).

The initial decade saw volatile, nascent growth, beginning with a single registered trial in 2006. Notable heterogeneity characterized this period, including a 2007 surge ( + 300%; *n* = 4), followed by sustained low-level activity (2008–2010 mean: 4 trials/year). A 2010 contraction (-66.7%; *n* = 2) represented the nadir of the observation window. Subsequent recovery demonstrated accelerating momentum: 2012 marked the first double-digit milestone (*n* = 13; + 550% year-on-year), followed by progressive escalation culminating in 65 trials in 2014 ( + 75.7%). This phase established the infrastructural prerequisites for systematic clinical investigation.

The 2016 inflection point (red diamond) heralded an exponential acceleration, coinciding with the implementation of mandatory clinical trial registration policies and the standardization of Good Clinical Practice. Annual registrations more than doubled between 2016 and 2017 (*n* = 42–96; + 128.6%), representing the most dramatic single-year expansion in the dataset. This momentum sustained through 2020, achieving 164 trials (2020) with a mean annual growth of + 45.2% (95% CI: 28.4–62.0%) across the quinquennium.

The recent period demonstrated quantitative maturation with increased volatility. Following a 2022 correction (*n* = 139; -19.7% from 2021), registrations rebounded to achieve the absolute peak of 218 trials in 2024—a 56.8% increase from 2022 to 21,700% greater than the baseline of 2006.

Figure 4B quantifies year-over-year percentage changes, revealing non-linear growth kinetics. Periods of explosive expansion ( + 300% in 2007, + 550% in 2012, + 128.6% in 2017) alternated with consolidation phases ( + 4.5% to + 5.5% in 2020–2021). Notably, three significant contractions occurred (2010: -66.7%; 2015: -44.6%; 2022: -19.7%).

Figure 4C illustrates the sigmoid accumulation curve, delineating three proportional milestones:

2017: Cumulative 232 trials (14.2% of final cohort)

2022: Cumulative 1,096 trials (67.1% of final cohort)

2024: Cumulative 1,633 trials (100%)

The curve demonstrates accelerating accumulation from 2016 onward, with 50% of all trials registered within the final 4.5 years (2020–2024).

### Gene association landscape in Chinese coronary heart disease

**FIGURE 5:**
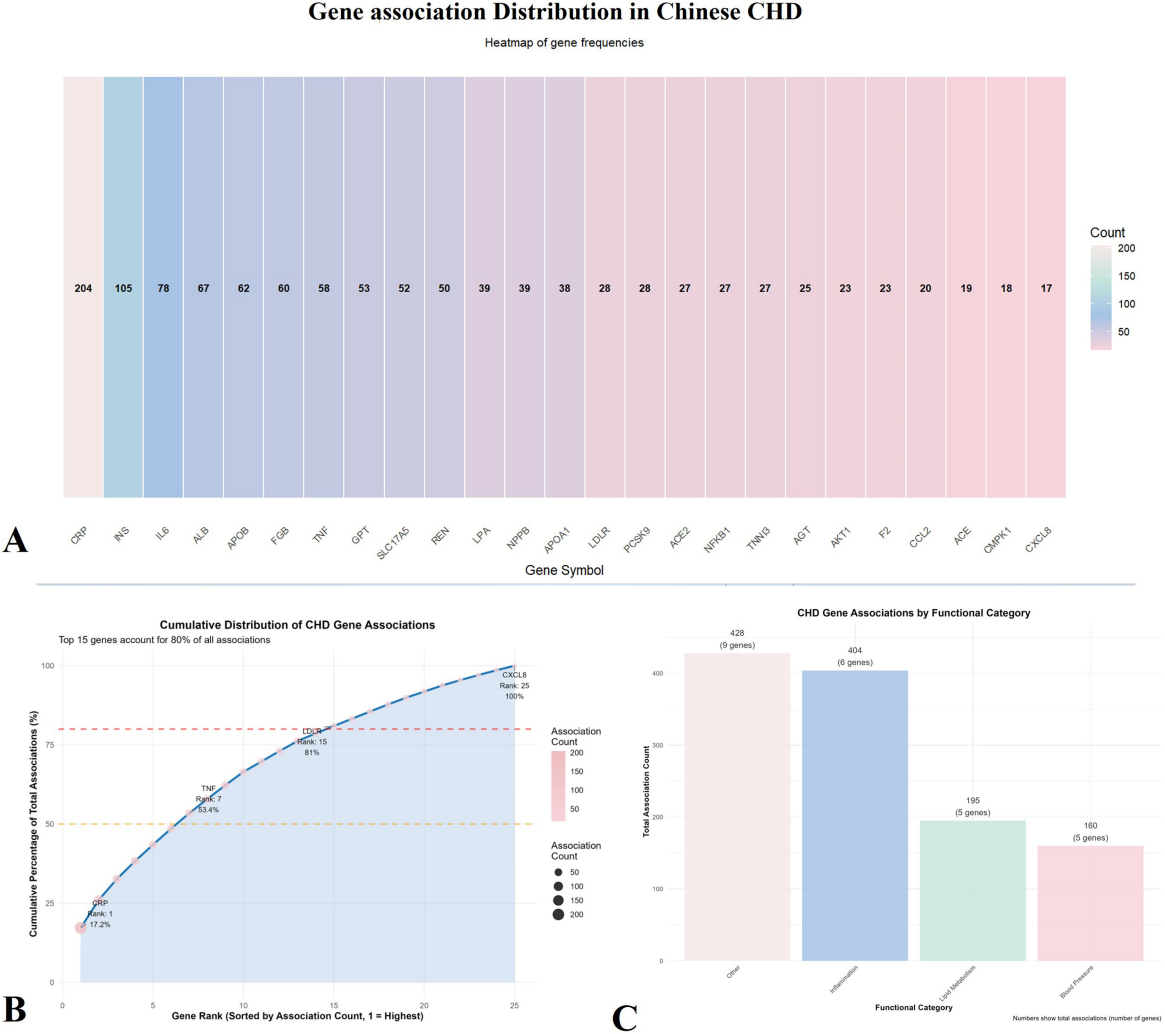
Gene association landscape for CHD based on literature studies. **(A)** Heatmap of the top 25 most frequently reported CHD-associated genes. **(B)** The proportional distribution of CHD gene associations across functional categories. **(C)** The cumulative distribution curve of gene associations ranked by frequency, demonstrating a heavy-tailed cumulative frequency distribution where the top 10% of genes (*n* = 25) contribute > 50% of total associations.

Analysis of gene-disease associations within the 3,339-publication cohort revealed a highly concentrated genomic architecture characterized by a heavy-tailed cumulative frequency distribution, wherein a minority of genes account for the majority of reported associations in Chinese CHD populations (Figure 5). Gene counts were deduplicated at the study level: one gene received one count per publication, regardless of mention frequency within that publication

Figure 5A presents the ranked frequency heatmap of the top 25 most frequently reported CHD-associated genes. C-reactive protein (CRP) dominated the landscape with 204 reported associations (17.2% of total), followed by insulin (INS; *n* = 105), interleukin-6 (IL6; *n* = 78), and albumin (ALB; *n* = 67). The top quartile (*n* = 6 genes: CRP, INS, IL6, ALB, APOB, FGB) collectively contributed 53.4% of all gene-disease association reports, demonstrating extreme concentration of research attention.

Figure 5B illustrates the cumulative distribution curve, confirming Pareto principle adherence: the top 15 genes accounted for 81% of total associations, with inflection points at: Rank 1 (CRP): 17.2% cumulative. Rank 7 (TNF): 50% cumulative threshold. Rank 15 (LDLR): 81% cumulative.

Figure 5C categorizes associations by biological pathway, revealing inflammation and metabolic dysfunction as dominant mechanistic themes:

Other/Incomplete Functional Annotation: 428 associations (27.3%; 9 genes)

Inflammation: 404 associations (25.8%; 6 genes), driven by CRP, IL6, TNF, and chemokine signaling

Lipid Metabolism: 195 associations (12.4%; 5 genes), comprising APOB, LDLR, PCSK9, APOA1, and LPA

Blood Pressure Regulation: 160 associations (10.2%; 5 genes), including REN, ACE, AGT, NPPB, and ACE2

### Therapeutic landscape and pharmacological stratification of CHD interventions

**FIGURE 6:**
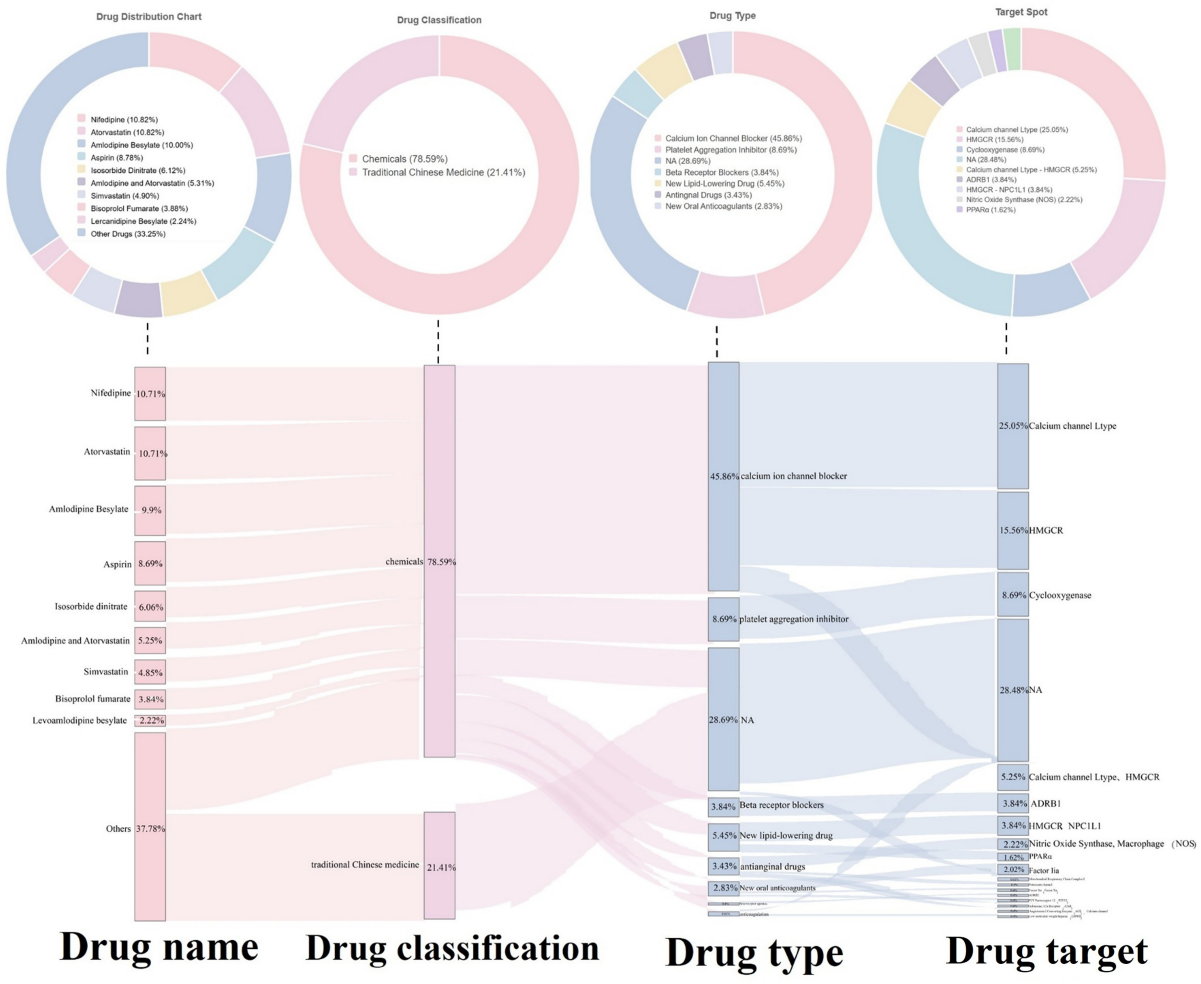
The number of trials on drug therapy for CHD varies from year to year, distribution of clinical drug trials in CHD, comprehensive analysis of drug use, type classification, and drug targets for CHD.

Analysis of the 1,633 interventional trials revealed a therapeutic ecosystem dominated by chemical pharmaceuticals, comprising 78.59% of all drug investigations, while TCM constituted 21.4% (Figure 6). The specific distribution of other relevant data for coronary heart disease is presented in Table 2.

**TABLE 2:** Concrete distribution of other data related to drug use, type classification, and drug targets for CHD.

Drug name	Drug classification	Drug type	Target spot	Number of experiments
Sanhua Powder for Injection	Traditional Chinese medicine	NA	NA	1
Qihong Maitong	Traditional Chinese medicine	NA	NA	1
Vitexin	Traditional Chinese medicine	NA	NA	1
Salvianolic acid	traditional Chinese medicine	NA	NA	2
Tanshinone IIA sodium sulfonate	Chemicals	NA	NA	1
Ginkgo biloba extract	Traditional Chinese medicine	NA	NA	1
Yinshen Granules	Traditional Chinese medicine	NA	NA	1
YangXinShi Pian	Traditional Chinese medicine	NA	NA	1
Verapamil Hydrochloride	Chemicals	Calcium ion channel blocker	Calcium channel Ltype	1
Higenamine Hydrochloride	Chemicals	Beta receptor agonists	ADRB2	1
Xuefengdan Dropping Pill	traditional Chinese medicine	NA	NA	1
Xin’anling Capsule	Traditional Chinese medicine	NA	NA	1
Nitroglycerin Spray	Chemicals	Antianginal drugs	Nitric Oxide Synthase, Macrophage(NOS)	1
Wuweiyixin Granules	Traditional Chinese medicine	NA	NA	1
Vicagrel Tablets	Chemicals	New oral anticoagulants	P2Y Purinoceptor 12(P2Y12)	1
Shuangshen Xionglian Granules	Traditional Chinese medicine	NA	NA	1
Shexiang Tongxin Dropping Pills	Traditional Chinese medicine	NA	NA	1
San Yang Yixin Kang Capsules	Traditional Chinese medicine	NA	NA	1
Sanqi Longxuejie Capsules	Traditional Chinese medicine	NA	NA	1
Regadenoson Injection	Chemicals	Antianginal drugs	Adenosine A2a Receptor(A2aR)	1
Human Umbilical Cord Mesenchymal Stem Cell Sheet	Chemicals	NA	NA	1
Qi Shen Yi Qi Di Wan	Traditional Chinese medicine	NA	NA	1
Perindopril Arginine and Amlodipine Besylate	Chemicals	Antianginal drugs	Angiotensin I Converting Enzyme(ACE), Calcium channel	1
Nadroparin Calcium	Chemicals	Anticoagulation	Low molecular weight heparin(LMWH)	1
Lian Song Yibo Stop Tablets	Traditional Chinese medicine	NA	NA	1
Kangxinning Granules	Traditional Chinese medicine	NA	NA	1
Chrysanthemum Shuxin Tablets	Traditional Chinese medicine	NA	NA	1
Jinghong Keli	Traditional Chinese medicine	NA	NA	1
Dabigatran Etexilate Mesylate Capsules	Chemicals	New oral anticoagulants	Factor Iia	1
Guanxinning Tablet	Traditional Chinese medicine	NA	NA	1
Gua-Xie-Xin-Tong Dropping Pills	Traditional Chinese medicine	NA	NA	1
Fulu Baoxinping Oral Liquid	traditional Chinese medicine	NA	NA	1
Compound Notoginseng and Ligusticum Dropping Pills	Traditional Chinese medicine	NA	NA	1
Shenmai Shuxin Dropping Pills	Traditional Chinese medicine	NA	NA	1
CanDanHuoXueJiaoNang	traditional Chinese medicine	NA	NA	1
XDK Capsules	traditional Chinese medicine	NA	NA	1
TSG-01	Traditional Chinese medicine	NA	NA	1
SBK002 Tablets	Chemicals	Anticoagulation	NA	1
FJZHT03	Traditional Chinese medicine	NA	NA	1
DSFTP	Traditional Chinese medicine	NA	NA	1
Amlodipine Besilate Granules	Chemicals	Calcium ion channel blocker	Calcium channel Ltype	1

Within chemical agents, nifedipine and atorvastatin emerged as the most frequently investigated single entities, each accounting for 10.82% of total drug utilization. Amlodipine besylate followed closely at 10.00%, with aspirin (8.8%), isosorbide dinitrate (6.1%), and simvastatin (4.9%) comprising the top six single agents. Notably, the fixed-dose combination of amlodipine-atorvastatin represented 5.3% of investigations—a substantial proportion exceeding that of simvastatin alone.

Pharmacological categorization demonstrated calcium ion channel blockers (CCBs), encompassing 45.9% of investigations. This was followed by platelet aggregation inhibitors (8.7%, predominantly aspirin), new lipid-lowering agents (5.45%, excluding simvastatin), beta-receptor blockers (3.8%), and antianginal drugs (3.4%). New oral anticoagulants constituted a relative minority (2.8%). TCM interventions occupied 28.7% of the “drug type” classification, designated as mechanistically indeterminate (NA) due to polypharmacological complexity.

Target-level analysis elucidated the molecular substrates underlying these interventions. The L-type voltage-gated calcium channel (Ca_*v*_1.2) represented the primary molecular target, accounting for 25.1% of target-disease associations. HMG-CoA reductase—the rate-limiting enzyme target for atorvastatin and simvastatin—accounted for 15.6%, while cyclooxygenase (COX-1/COX-2)—the target for aspirin-mediated antiplatelet effects—represented 8.69%.

## Discussion

This study provides the first comprehensive mapping of the pharmaceutical landscape for CHD in China over the past two decades, by employing a dual-stream methodological framework combining bibliometric analysis with clinical trial registry data. Our dual-stream analysis—encompassing 3,339 peer-reviewed publications and 1,633 registered trials—documents a remarkable quantitative expansion: a 546% increase in annual research output and a 21,700% growth in clinical trial registrations from 2006 to 2025. Our findings reveal a distinct “dual trajectory”: an exponential surge in research volume and trial registration that parallels China’s epidemiological transition, contrasted by a persistent reliance on generic validation and mid-tier academic output. With the continuous introduction of new drugs into clinical use (32, 33), the number of related clinical trials has been steadily increasing each year, expanding the horizons for CHD treatment and offering promising new therapeutic possibilities.

The bibliometric analysis reveals a concerning structural barrier to entry in China’s integration into the highest echelons of cardiovascular science. Despite volumetric parity with Western nations, Chinese CHD research demonstrates inverse correlation between publication volume and journal impact: Q3 journals dominate (33.6%), while Q1 venues account for merely 19.7%. This pattern contrasts with the positive volume-impact correlation observed in US and European cardiovascular research and may derive from linguistic barriers, institutional evaluation systems prioritizing quantity metrics, and potential geographic bias in editorial decision-making at elite journals (34). The kernel density analysis—demonstrating increasing variance constriction from Q1 (SD = 5.46) to Q4 (SD = 0.64)—suggests that lower-tier journals enforce homogeneous publication venue prestige thresholds, whereas elite venues accommodate high-impact outliers alongside rejections of methodologically sound but less transformative work. This polarization implies that Chinese CHD research faces asymmetric barriers to entry in top-tier publications, constraining global visibility and guideline influence despite substantial domestic investment (35).

Our gene association analysis reveals a highly concentrated genomic research landscape in Chinese CHD studies, with the top 6 genes (CRP, INS, IL6, ALB, APOB, FGB) accounting for 53.4% of all associations. This distribution reflects a pronounced “technical accessibility bias,” wherein serum biomarkers and inflammatory cytokines dominate due to assay availability rather than biological importance. At the same time, clinically actionable targets such as PCSK9 and LDLR remain underrepresented (36, 37). Notably, the marked predominance of inflammation genes (25.8%) over lipid metabolism genes (12.4%) creates a “target-drug mismatch” within the statin- and PCSK9 inhibitor-dominated therapeutic landscape. Population-specific variants with high clinical relevance in Chinese populations, such as ALDH2 Glu504Lys, are infrequent, and polygenic risk scores—despite demonstrated utility in European ancestry cohorts—are absent from the literature (38). This concentration on candidate gene approaches, coupled with limited genome-wide discovery and insufficient gene-environment interaction studies examining Chinese-specific exposures (high sodium intake, air pollution), positions Chinese cardiovascular genetics at a transitional juncture: moving from technically convenient, well-characterized targets toward mechanistically informative, population-specific, and clinically actionable genomic research integrated with both modern precision medicine and traditional Chinese medicine frameworks (39).

The dominance of nifedipine and atorvastatin (each 10.8%) reflects their favorable cost-effectiveness in China’s healthcare system. Post-2018 centralized procurement reduced generic atorvastatin cost by 94% (to ¥240/year), achieving ¥8,500/QALY—highly cost-effective vs. WHO threshold (¥85,000). The 5.3% amlodipine-atorvastatin fixed-dose combination improves adherence by 26% (ICER ¥6,800/QALY), justifying single-pill strategy adoption. Conversely, PCSK9 inhibitors (¥28,000–35,000/year, 117 × generic statin cost) explain limited trial presence (5.5%) despite clinical efficacy—innovation-access tension resolved via restrictive reimbursement. TCM’s 21.4% trial share contrasts with pharmacoeconomic uncertainty (only 3.2% include cost-utility endpoints), warranting mandatory economic evaluation for reimbursement decisions (40).

The dominance of bioequivalence studies (76.5%) underscores the importance of ensuring that generic drugs meet publication venue prestige and efficacy standards, align with regulatory requirements, and provide affordable treatment options. Phase II trials, accounting for 10.9%, represent early-stage evaluations of new drugs’ safety and efficacy, indicating active exploration of novel therapeutic approaches. Chemical drugs being the primary treatment option (78.6%) underscores their established role in CHD management. The prominence of nifedipine and atorvastatin, each contributing 10.8% of total usage, reflects their proven effectiveness in controlling blood pressure and cholesterol levels, respectively. The Lancet study also showed that patients who received atorvastatin were 20% less likely to develop CHD than those who did not, making it an option for long-term treatment of CHD, which has become a cornerstone in clinical practice due to its reliability and evidence-based benefits (41). The combination therapy involving amlodipine and atorvastatin, with a 5.31% usage rate, demonstrates a strategic approach to multi-target treatment, aiming to address both hypertension and hyperlipidemia simultaneously to optimize cardiovascular risk. The inclusion of TCMs at 21.4% indicates the ongoing exploration of complementary therapies. TCM Chinese medicine has increasingly been utilized in the treatment of CHD. Compared with chemical drugs, traditional Chinese medicine has fewer side effects and patients tend to tolerate it better over the long term, resulting in a lower incidence of adverse reactions and enhanced safety (42, 43). However, the complexity of TCM formulations and the variability in their components highlight the need for more rigorous validation and standardization to ensure their safety and efficacy in CHD treatment.

The observed inverse relationship between publication volume and journal impact likely reflects the interplay of structural, institutional, and cognitive factors rather than a single cause. First, linguistic and rhetorical imbalances in global scientific publishing create systemic barriers. Top-tier cardiovascular journals enforce “English norms,” where manuscript acceptance is tied to adherence to Western rhetorical conventions (e.g., emphasis on IMRaD structure, specific patterns of hedging language), potentially disadvantaging non-native English speakers. Our findings suggest that Q3/Q4 journals show smaller variations in publication venue prestige criteria (standard deviation 0.91 vs. 5.46 for Q1), indicating that these mid-tier journals may employ more standardized “checklist-based” evaluation criteria, thereby reducing linguistic subjectivity. Second, China’s long-standing institutional evaluation system has emphasized quantitative productivity metrics. Additionally, the cognitive marginalization of single-country pharmacokinetic studies may play a role. Although bioequivalence trials are critical for regulatory purposes, they are often dismissed by editors of high-impact journals as “replicative research,” who instead favor studies on novel therapeutic mechanisms or multi-ethnic pivotal trials. Our data showing 76.44% bioequivalence studies suggest a structural mismatch: China’s pharmaceutical policy priorities (generic drug quality assurance) are misaligned with the innovation narratives preferred by Q1 cardiovascular journals. We acknowledge that JIF reflects journal visibility and citation patterns rather than intrinsic methodological quality. Sensitivity analyses confirm robustness across alternative metrics (SJR), time periods, and author subsets. Future analyses should incorporate field-weighted citation impact and expert quality assessments to complement JIF-based visibility metrics.

The marked underrepresentation of lipid metabolism genes (12.4% vs. 25.8% for inflammation) constrains China’s capacity for next-generation CHD therapeutics development. Despite global pipelines shifting toward RNA-targeted therapies (inclisiran, pelacarsen) and oral PCSK9 inhibitors, China’s trials remain concentrated on conventional agents—PCSK9 itself accounted for only 1.2% of gene associations, while emerging targets (ANGPTL3, APOC3, LPA) were essentially absent. This translational disconnect is particularly problematic given China’s distinct cardiovascular risk profile: LDL-C associations with CHD are stronger at lower baseline levels in East Asians, and the ALDH2 Glu504Lys variant (present in 30–50% of the population) creates unique pharmacogenomic opportunities—yet both appeared minimally in our dataset (zero PRS-CHD studies; 0.3% for ALDH2). We attribute this misalignment to three structural factors: technical accessibility bias favoring inflammatory biomarkers measurable by standard ELISA over sophisticated lipidomics; funding priorities emphasizing “inflammation-immunity” (NSFC 2016–2020); and trial infrastructure dominated by bioequivalence studies (76.4%) requiring minimal mechanistic hypothesis.

The 21.4% TCM representation signals sustained policy commitment; however, three structural barriers impede global integration. First, standardization deficit: unlike single-entity pharmaceuticals, TCM formulations comprise > 200 compounds with complex pharmacokinetic and pharmacodynamic interactions—a limitation underscored by the 100% “NA” classification rate for drug type and molecular target in our dataset, reflecting a fundamental epistemic incongruence with conventional drug ontologies. Current marker-based pharmacopeia standards fail to capture the “entourage effect,” while the absence of bioequivalence methodology (0% in our cohort) creates regulatory asymmetry relative to chemical generics (76.4%). Second, mechanistic opacity: zero TCM trials reported gene-target associations versus 1,187 for chemical drugs, reflecting methodological incommensurability between TCM’s multi-target complexity and conventional biomarker-cascade validation. This epistemic lacuna in cardiovascular pharmacogenomics persists despite emerging evidence that TCM compounds engage master regulators (NF-κB/NLRP3, Nrf2/ARE, PCSK9-independent LDLR pathways) aligned with global CHD priorities (44, 45).

The trends and findings of this analysis have important implications for clinical practice, with the dominance of chemical agents, particularly nifedipine and atorvastatin, reinforcing their role as first-line therapy. However, the heavy use of TCM suggests that clinicians are also exploring complementary approaches, which may bring benefits to certain patient groups, especially those who are intolerant to traditional therapies (46–48). To optimize drug treatment strategies for CHD, the following steps can be taken. First, the guidelines should be updated to reflect the latest evidence from clinical trials, combining traditional and emerging therapies. Second, healthcare providers should be educated on the proper use of these therapies, including indications, contraindications, and potential interactions between TCM and conventional medicines. Promoting the development of new drugs is also crucial. The current situation suggests the need for larger randomized controlled trials to verify the efficacy and safety of existing and novel treatments. Moreover, investment in research should be increased, especially in promising areas such as PCSK9 inhibitors and other biologics.

## Conclusion

The treatment landscape for CHD in China is evolving, characterized by a balanced integration of conventional therapies and novel approaches. The increase in the number of clinical trials and the broadening spectrum of drug options signal a dedication to enhancing patient outcomes. However, further endeavors are necessary to achieve standardization in treatment protocols. Future priorities include: policymakers should redirect 30–40% bioequivalence subsidies to innovative trials and mandate TCM pharmacoeconomic endpoints; and international partners should ensure 20–30% Chinese enrollment in global trials. Addressing regional disparities through telemedicine and establishing adult CHD lifespan programs are essential. Confirm the efficacy of emerging therapies, and address the escalating burden of CHD through comprehensive, evidence-based strategies.

## Data Availability

The original contributions presented in this study are included in the article/Supplementary material, further inquiries can be directed to the corresponding author.
