# Supplementary material for: Clinical analysis of drug treatment trends for coronary heart disease in China
Source: Front Med (Lausanne). 2026 Jun 9;13:1809277. doi: 10.3389/fmed.2026.1809277 (PMC13286841; doi:10.3389/fmed.2026.1809277)
Supplement: Supplementary file 1 [file Data_Sheet_1.docx]

|  |  |  |  |
| --- | --- | --- | --- |
| By July 2025 | PubMed | (China[Title/Abstract]) AND (((((Coronary Diseases[Title/Abstract]) OR (Disease, Coronary[Title/Abstract])) OR (Diseases, Coronary[Title/Abstract])) OR (Coronary Heart Disease[Title/Abstract])) OR ("Coronary Disease"[Mesh])) | 2504 |
|  | Embase | ('coronary disease'/exp OR 'coronary diseases' OR (coronary AND ('disease'/exp OR disease)) OR 'disease, coronary':ab,ti OR 'coronary diseases':ab,ti OR 'diseases, coronary':ab, ti) AND 'china':ab,ti | 7413 |
|  | Web of Science | (China[Title/Abstract]) AND (((((Coronary Diseases[Title/Abstract]) OR (Disease, Coronary[Title/Abstract])) OR (Diseases, Coronary[Title/Abstract])) OR (Coronary Heart Disease[Title/Abstract])) OR ("Coronary Disease"[Mesh])) AND ('major clinical study'/de OR 'randomized controlled trial'/de) | 303 |
|  | Scopus | (China[Title/Abstract]) AND (((((Coronary Diseases[Title/Abstract]) OR (Disease, Coronary[Title/Abstract])) OR (Diseases, Coronary[Title/Abstract])) OR (Coronary Heart Disease[Title/Abstract])) OR ("Coronary Disease"[Mesh])) | 1812 |

**Supplementary Table 1: Search term information**

| Drug name | Drug classification | Drug type | Drug target |  | Number of experiments |
| --- | --- | --- | --- | --- | --- |
| Nifedipine | chemicals | calcium ion channel blocker | Calcium channel Ltype | | 53 |
| Atorvastatin | chemicals | calcium ion channel blocker | HMGCR |  | 53 |
| Amlodipine Besylate | chemicals | calcium ion channel blocker | Calcium channel Ltype | | 48 |
| Aspirin | chemicals | platelet aggregation inhibitor | Cyclooxygenase |  | 42 |
| Isosorbide dinitrate | chemicals | NA | NA |  | 29 |
| Amlodipine and Atorvastatin | chemicals | calcium ion channel blocker | Calcium channel Ltype、HMGCR | | 25 |
| Simvastatin | chemicals | calcium ion channel blocker | HMGCR |  | 22 |
| Bisoprolol fumarate | chemicals | Beta receptor blockers | ADRB1 |  | 17 |
| Levoamlodipine besylate | chemicals | calcium ion channel blocker | Calcium channel Ltype | | 11 |
| Rosuvastatin, ezetimibe | chemicals | New lipid-lowering drug | HMGCR、NPC1L1 |  | 10 |
| Ezelimabresuvastatin | chemicals | New lipid-lowering drug | HMGCR、NPC1L1 |  | 9 |
| Shen-Hua Capsules | traditional Chinese medicine | | NA |  | 9 |
| Nitroglycerin | chemicals | antianginal drugs | Nitric Oxide Synthase, Macrophage（NOS） | | 8 |
| Fenofibrate | chemicals | New lipid-lowering drug | PPARα |  | 8 |
| Dabigatran etexilate | chemicals | New oral anticoagulants | Factor Iia |  | 8 |
| Jiashen Tablets | traditional Chinese medicine | | NA |  | 5 |
| Danshensu sodium | traditional Chinese medicine | | NA |  | 5 |
| Amlodipine besylate | chemicals | calcium ion channel blocker | Calcium channel Ltype | | 5 |
| Xinmaikang capsules | traditional Chinese medicine | | NA |  | 4 |
| Ginkgolide | traditional Chinese medicine | | NA |  | 3 |
| Yangshenguo Guanxin Tablets | traditional Chinese medicine | | NA |  | 3 |
| Plug heart-saving tablets | traditional Chinese medicine | | NA |  | 3 |
| Longya Guanshinkang Capsules | traditional Chinese medicine | | NA |  | 3 |
| XTR004 injection | chemicals | NA | Mitochondrial Respiratory Chain Complex I | | 3 |
| Yanhuang Xiaoxintong Capsules | traditional Chinese medicine | | NA |  | 2 |
| Xuesaitong enteric-coated tablets | traditional Chinese medicine | | NA |  | 2 |
| Xuefu Zhuyu Capsules | traditional Chinese medicine | | NA |  | 2 |
| Xin Shukan Tablets | traditional Chinese medicine | | NA |  | 2 |
| Nicolandil | chemicals | antianginal drugs | Potassium channel |  | 2 |
| Levoamlodipine maleate | chemicals | calcium ion channel blocker | Calcium channel Ltype | | 2 |
| Rivaroxaban | chemicals | New oral anticoagulants | Factor Xa（Factor Xa） | | 2 |
| Huang Yang | traditional Chinese medicine | | NA |  | 2 |
| Honghua Dropping Pills | traditional Chinese medicine | | NA |  | 2 |
| Exinone tablets | traditional Chinese medicine | | NA |  | 2 |
| Cyclovirobuxine D Hydrochloride | traditional Chinese medicine | | NA |  | 2 |
| Sanhua Powder for Injection | traditional Chinese medicine | | NA |  | 1 |
| Qihong Maitong | traditional Chinese medicine | | NA |  | 1 |
| Vitexin | traditional Chinese medicine | | NA |  | 1 |
| Salvianolic acid | traditional Chinese medicine | | NA |  | 2 |
| Tanshinone IIA sodium sulfonate | chemicals | NA | NA |  | 1 |
| Ginkgo biloba extract | traditional Chinese medicine | | NA |  | 1 |
| Yinshen Granules | traditional Chinese medicine | | NA |  | 1 |
| YangXinShi Pian | traditional Chinese medicine | | NA |  | 1 |
| Verapamil Hydrochloride | chemicals | calcium ion channel blocker | Calcium channel Ltype | | 1 |
| Higenamine Hydrochloride | chemicals | Beta receptor agonists | ADRB2 |  | 1 |
| Xuefengdan Dropping Pill | traditional Chinese medicine | | NA |  | 1 |
| Xin'anling Capsule | traditional Chinese medicine | | NA |  | 1 |
| Nitroglycerin Spray | chemicals | antianginal drugs | Nitric Oxide Synthase, Macrophage（NOS） | | 1 |
| Wuweiyixin Granules | traditional Chinese medicine | | NA |  | 1 |
| Vicagrel Tablets | chemicals | New oral anticoagulants | P2Y Purinoceptor 12（P2Y12） | | 1 |
| Shuangshen Xionglian Granules | traditional Chinese medicine | | NA |  | 1 |
| Shexiang Tongxin Dropping Pills | traditional Chinese medicine | | NA |  | 1 |
| San Yang Yixin Kang Capsules | traditional Chinese medicine | | NA |  | 1 |
| Sanqi Longxuejie Capsules | traditional Chinese medicine | | NA |  | 1 |
| Regadenoson Injection | chemicals | antianginal drugs | Adenosine A2a Receptor（A2aR） | | 1 |
| Human Umbilical Cord Mesenchymal Stem Cell Sheet | chemicals | NA | NA |  | 1 |
| Qi Shen Yi Qi Di Wan | traditional Chinese medicine | | NA |  | 1 |
| Perindopril Arginine and Amlodipine Besylate | chemicals | antianginal drugs | Angiotensin I Converting Enzyme（ACE）、Calcium channel | | 1 |
| Nadroparin Calcium | chemicals | anticoagulation | Low molecular weight heparin（LMWH） | | 1 |
| Lian Song Yibo Stop Tablets | traditional Chinese medicine | | NA |  | 1 |
| Kangxinning Granules | traditional Chinese medicine | | NA |  | 1 |
| Chrysanthemum Shuxin Tablets | traditional Chinese medicine | | NA |  | 1 |
| Jinghong Keli | traditional Chinese medicine | | NA |  | 1 |
| Dabigatran Etexilate Mesylate Capsules | chemicals | New oral anticoagulants | Factor Iia |  | 1 |
| Guanxinning Tablet | traditional Chinese medicine | | NA |  | 1 |
| Gua-Xie-Xin-Tong Dropping Pills | traditional Chinese medicine | | NA |  | 1 |
| Fulu Baoxinping Oral Liquid | traditional Chinese medicine | | NA |  | 1 |
| Compound Notoginseng and Ligusticum Dropping Pills | traditional Chinese medicine | | NA |  | 1 |
| Shenmai Shuxin Dropping Pills | traditional Chinese medicine | | NA |  | 1 |
| CanDanHuoXueJiaoNang | traditional Chinese medicine | | NA |  | 1 |
| XDK Capsules | traditional Chinese medicine | | NA |  | 1 |
| TSG-01 | traditional Chinese medicine | | NA |  | 1 |
| SBK002 Tablets | chemicals | anticoagulation | NA |  | 1 |
| FJZHT03 | traditional Chinese medicine | | NA |  | 1 |
| DSFTP | traditional Chinese medicine | | NA |  | 1 |
| Amlodipine Besilate Granules | chemicals | calcium ion channel blocker | Calcium channel Ltype | | 1 |

**Supplement Table 2: the relevant situation of the trial data**

| **TCM Category** | **Representative Examples** | **Trial Count** | **Percentage of TCM Trials** | **Rationale for NA Classification** |
| --- | --- | --- | --- | --- |
| **Cardiovascular patent medicines** | Compound Danshen Dripping Pills, Shexiang Baoxin Pills | 89 | 29.0% | Polyherbal formulations (>3 botanicals); targets not individually validated |
| **Single-herb extracts** | Salvianolic acid, Tanshinone IIA, Ginkgo biloba extract | 67 | 21.8% | Multiple active constituents with pleiotropic effects |
| **Injectables** | Shengmai Injection, Danhong Injection | 54 | 17.6% | Complex mixtures; batch-to-batch variability in marker compounds |
| **Oral decoctions/modified formulas** | Guanxinning decoctions, personalized prescriptions | 62 | 20.2% | Customized compositions; no fixed formula |
| **Health products/functional foods** | Coenzyme Q10 combinations, herbal supplements | 35 | 11.4% | Regulatory classification ambiguous; not standardized pharmaceuticals |
| **Total TCM trials** | — | 307 | 100% | — |

**Supplementary Table 3: TCM Intervention Categories**
